# Increase in serotype 19A prevalence and amoxicillin non-susceptibility among paediatric *Streptococcus pneumoniae *isolates from middle ear fluid in a passive laboratory-based surveillance in Spain, 1997-2009

**DOI:** 10.1186/1471-2334-11-239

**Published:** 2011-09-12

**Authors:** Asunción Fenoll, Lorenzo Aguilar, Maria-Dolores Vicioso, Maria-Jose Gimenez, Olga Robledo, Juan-Jose Granizo

**Affiliations:** 1 Spanish Reference Pneumococcal Laboratory, Inst. Salud Carlos III, ctra. Majadahonda-Pozuelo Km. 2, 28220 Majadahonda, Madrid, Spain; 2 Microbiology Dept., School of Medicine, Univ. Complutense, Avda. Complutense s/n, 28040 Madrid, Spain; 3 Grana Datos, Isla de Arosa 11, 28223 Pozuelo de Alarcón, Madrid, Spain

**Keywords:** Susceptibility, serotype 19A, middle ear isolates, non-susceptibility time trends, surveillance

## Abstract

**Background:**

Conjugate vaccines, such as the 7-valent conjugate vaccine (PCV7), alter serotype nasopharyngeal carriage, potentially increasing cases of otitis media by non-vaccine serotypes.

**Methods:**

All paediatric middle ear fluid (MEF) isolates received in the Spanish Reference Laboratory for Pneumococci through a passive, laboratory-based surveillance system from January 1997 to June 2009 were analysed. Data from 1997 to 2000 were pooled as pre-vaccination period. Trends over time were explored by linear regression analysis.

**Results:**

A total of 2,077 isolates were analysed: 855 belonging to PCV7 serotypes, 466 to serotype 19A, 215 to serotype 3, 89 to serotype 6A and 452 to other serotypes (< 40 isolates each). Over time, there has been a decreasing trend for PCV7 serotypes (R^2 ^= 0.944; p < 0.001, with significant decreasing trends for serotypes 19F, 14, 23F and 9V), and increasing trends for serotype 19A (R^2 ^= 0.901; p < 0.001), serotype 3 (R^2 ^= 0.463; p = 0.030) and other non-PCV7 serotypes (R^2 ^= 0.877; p < 0.001), but not for serotype 6A (R^2 ^= 0.311; p = 0.094). Considering all isolates, amoxicillin non-susceptibility showed an increasing trend (R^2 ^= 0.528; p = 0.017). Regarding serotype 19A, increasing trends in non-susceptibility to penicillin (R^2 ^= 0.726; p = 0.001), amoxicillin (R^2 ^= 0.804; p < 0.001), cefotaxime (R^2 ^= 0.546; p = 0.005) and erythromycin (R^2 ^= 0.546; p = 0.009) were found, with amoxicillin non-susceptibility firstly detected in 2003 (7.4%) and increasing up to 38.0% in 2009. In PCV7 serotypes (which prevalence decreased from 70.7% during 1997-2000 to 10.6% in 2009) amoxicillin non-susceptibility rates showed an increasing trend (R^2 ^= 0.702; p = 0.002). However, overall, amoxicillin non-susceptibility (≈25% in 2008-9) could be mainly attributed to serotype 19A (> 35% isolates) since PCV7 strains represented < 11% of total clinical isolates.

**Conclusions:**

In contrast to reports on invasive pneumococcal strains, in MEF isolates the reduction in the prevalence of PCV7 serotypes was not associated with decreases in penicillin/erythromycin non-susceptibility. The high prevalence of serotype 19A among paediatric MEF isolates and the amoxicillin non-susceptibility found in this serotype are worrisome since amoxicillin is the most common antibiotic used in the treatment of acute otitis media. These data suggest that non-PCV7 serotypes (mainly serotype 19A followed by serotypes 3 and 6A) are important etiological agents of acute otitis media and support the added value of the broader coverage of the new 13-valent conjugate vaccine.

## Background

Acute otitis media (AOM) is the most common infection in children following pneumococcal colonization of the upper respiratory tract [1], and the most common reason for which antibiotics are prescribed in children [2]. *Streptococcus pneumoniae *is worldwide identified in 30-60% cases of AOM [3,4]. In recent decades the emergence of pneumococcal strains exhibiting non-susceptibility to penicillin, especially in middle ear fluid isolates [5-7], has evolved into a global problem because non-susceptibility to penicillin is associated with decreases in the intrinsic activity of other oral β-lactams.

Among the 93 pneumococcal serotypes [8], the seven most commonly found in the US causing invasive disease in children are targeted by the 7-valent pneumococcal conjugate vaccine (PCV7) [9]. PCV7 is available in Spain (approx. 45 million inhabitants) since October 2001, but only in the private market for children under 2 years of age, with a vaccination schedule of 2-, 4-, 6-month primary series plus a fourth dose at 15-18 months. Although the vaccine use increased from 2002 onwards, the reported vaccine coverage in 2006 was below 50% (assuming complete vaccination schedules) [10]. In October 2006, the Autonomous Region of Madrid (approx. 6 million inhabitants) approved its inclusion in the childhood vaccination calendar with the vaccination schedule stated above. Despite the irregular regional PCV7 uptake in Spain, vaccination has resulted in a decrease in PCV7 serotypes among invasive isolates from children and adults (due to herd effect). This reduction has been associated with a decrease in the proportion of isolates non-susceptible to penicillin [11-13]. However, as in other countries, these changes were associated with an increase in the prevalence of non-PCV7 serotypes causing invasive disease [11].

Conjugate vaccines protect against nasopharyngeal carriage of serotypes included in the vaccine [14]. This ecological niche can then be filled by other serotypes [15], with subsequent potential increases in otitis media by non-vaccine serotypes [14,16].

This study focuses on pneumococcal isolates from middle ear fluid received in the Spanish Reference Laboratory for Pneumococci (SRLP) from hospitals located all over Spain, and analyses changes in serotype distribution and antibiotic susceptibility in the period 1997-2009.

## Methods

All paediatric (≤ 14 years of age) *S. pneumoniae *isolates from middle ear fluid received in the SRLP from hospitals located all over the 17 Autonomous Regions in Spain from January 1997 to June 2009 were analysed. Isolates had been sent on a voluntary basis through a passive, laboratory-based surveillance system. All MEF isolates in global and the subgroups of PCV7 and 19A isolates were distributed per age group (< 2 years, 2-4 years and 5-14 years). Data from 1997 to 2000 were pooled as pre-vaccination period. Isolates were serotyped by Quellung reaction [17] and/or Dot blot assay [18]. Susceptibility to penicillin, amoxicillin, cefotaxime and erythromycin was determined by agar dilution using Mueller-Hinton agar (Difco Laboratories, Detroit, MI, USA) supplemented with 5% sheep blood (Biomedics, Madrid, Spain) as culture media. Plates were incubated under 5% CO_2 _atmosphere as recommended by the Clinical and Laboratory Standards Institute [19]. *S. pneumoniae *ATCC 6303 and *S. pneumoniae *ATCC 49619 plus six clinical isolates were used as quality controls [20]. Current non-susceptibility breakpoints for oral penicillin (MIC ≥ 0.12 μg/ml), amoxicillin (MIC ≥ 4 μg/ml), cefotaxime (MIC ≥ 2 μg/ml; non-meningitis) and erythromycin (MIC ≥ 0.5 μg/ml) [19] were considered.

Trends along time (1997-2000 to 2009) of serotype prevalence and non-susceptibility rates were explored by regression analysis (SPSS v14 programme, SPSS Inc, Chicago IL) with time as independent variable. Linear regression analysis was used, based on highest goodness of fit.

## Results

Table 1 shows the annual total number of paediatric pneumococcal isolates received in the SRLP from January 1997 to June 2009, and the number (and percentage) of paediatric isolates from middle ear fluid. As shown, no great variations in the annual percentage of isolates from middle ear fluid among all paediatric isolates received in the SRLP were found over time. Of the 2,077 middle ear fluid isolates from children ≤ 14 years of age received in the study period (481 from the period 1997-2000 and 1,596 from 2001 on), 855 isolates belonged to PCV7 serotypes, 466 to serotype 19A, 215 to serotype 3, 89 to serotype 6A and 452 to other serotypes (with < 40 isolates each). Table 1 also shows the annual distribution per age group (< 2 years, 2-4 years and 5-14 years) of total MEF isolates, and PCV7 and 19A isolates among them. Significant (p < 0.001) decreasing trends (R^2 ^≥ 0.851) were found for the pooled PCV7 serotypes in the three age groups, with β ranging from -0.923 to -0.952. In contrast, significant increasing trends were found for serotype 19A mainly in the < 2 years age group (R^2 ^= 0.940; β = 0.969; p < 0.001) and, to a lesser extent, in the 2-4 years group (R^2 ^= 0.633; β = 0.796; p < 0.006), but not in the 5-14 years group.

**Table 1:** Annual total number of paediatric isolates (invasive + non-invasive) received by the SRLP, total no. (%) of paediatric middle ear fluid isolates (MEF), and among them, no. (%) of PCV7 and 19A isolates.

	97-00	2001	2002	2003	2004	2005	2006	2007	2008	2009
**Total no**.	2342	758	874	802	1196	1374	1136	1326	1291	749
**MEF [n (%)]**	481 (20.5)	210 (27.7)	171 (19.6)	159 (19.8)	180 (15.1)	166 (12.1)	176 (15.5)	173 (13.0)	220 (17.0)	141 (18.8)
< 2 yrs.	253 (52.6)	121 (57.6)	81(47.4)	69 (43.4)	81 (45.0)	91 (54.8)	97 (55.1)	95 (54.9)	153 (69.5)	86 (61.0)
2-4 yrs.	68 (14.1)	43 (20.5)	49 (28.7)	34 (21.4)	46 (25.6)	26 (15.7)	50 (28.4)	51 (29.5)	44 (20.0)	35 (24.8)
5-14 yrs.	160 (33.3)	46 (21.9)	41 (24.0)	56 (35.2)	53 (29.4)	49 (29.5)	29 (16.5)	27 (15.6)	23 (10.5)	20 (14.2)
PCV-7 [n (%)]*	340 (70.7)	132 (62.9)	109 (63.7)	77 (48.4)	64 (35.6)	40 (24.1)	33 (18.8)	23 (13.3)	22 (10.0)	15 (10.6)
< 2 yrs.	186 (54.7)	86 (65.2)	59 (54.1)	37 (48.1)	35 (54.7)	23 (57.5)	11 (33.3)	10 (43.5)	15 (68.2)	10 (66.7)
2-4 yrs.	54 (15.9)	20 (15.2)	30 (27.5)	13 (16.9)	16 (25.0)	7 (17.5)	12 (36.4)	10 (43.5)	5 (22.7)	2 (13.3)
5-14 yrs.	100 (29.4)	26 (19.7)	20 (18.3)	27 (35.1)	13 (20.3)	10 (25.0)	10 (30.3)	3 (13.0)	2 (9.1)	3 (20.0)
19A [n (%)]*	32 (6.7)	20 (9.5)	21 (12.3)	27 (17.0)	38 (21.1)	53 (31.9)	55 (31.3)	67 (38.7)	103 (46.8)	50 (35.5)
< 2 yrs.	18 (56.3)	14 (70.0)	9 (42.9)	14 (51.9)	20 (52.6)	35 (66.0)	41 (74.5)	48 (71.6)	80 (77.7)	42 (84.0)
2-4 yrs.	3 (9.4)	3 (15.0)	4 (19.0)	6 (22.2)	7 (18.4)	5 (9.4)	10 (18.2)	11 (16.4)	19 (18.4)	7 (14.0)
5-14 yrs.	11 (34.4)	3 (15.0)	8 (38.1)	7 (25.9)	11 (28.9)	13 (24.5)	4 (7.3)	8 (11.9)	4 (3.9)	1 (2.0)

Distribution [no. (%)] per-age group (< 2 years, 2-4 years and 5-14 years) of total MEF isolates and of PCV-7 and 19A isolates among them.

*Percentages among MEF isolates

**Figure 1:**
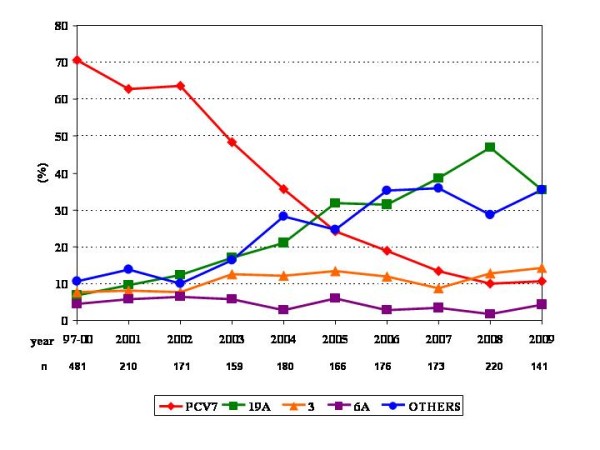
**Annual prevalence of isolates belonging to PCV7 serotypes (as a group), serotype 19A, serotype 3, serotype 6A and other grouped non-PCV7 serotypes**.

Figure 1 shows percentages over time of all middle ear isolates distributed by serotype including PCV7 pooled serotypes, serotypes 19A, 3, 6A and other grouped serotypes. Our results show a significant decreasing trend for PCV7 serotypes (R^2 ^= 0.944; β = -0.972; p < 0.001), and significant increasing trends for serotype 19A (R^2 ^= 0.901; β = 0.949; p < 0.001) and, to a lesser extent, for serotype 3 (R^2 ^= 0.463; β = 0.681; p = 0.030). No significant trend was found for serotype 6A (R^2 ^= 0.311; β = -0.558; p = 0.094). In addition, other pooled non-PCV7 serotypes (39 serotypes in total) exhibited a significant increasing trend (R^2 ^= 0.806 β = 0.898; p < 0.001).

**Figure 2:**
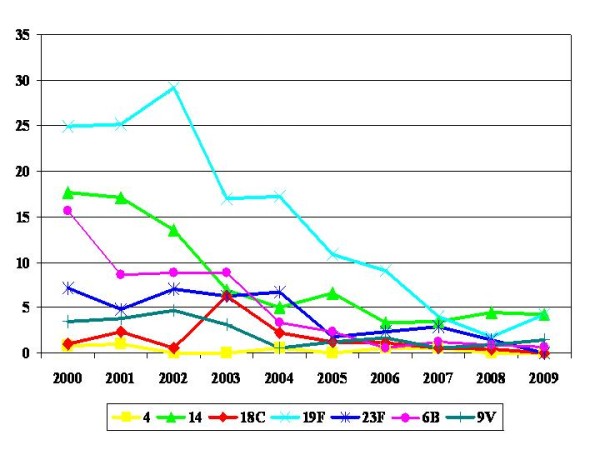
**Annual prevalence of isolates belonging to serotypes included in PCV7**.

Figure 2 shows the percentages of isolates belonging to serotypes included in PCV7 over time. Significant decreasing trends were found for serotypes 19F (322 isolates; R^2 ^= 0.875; β = -0.943; p < 0.001), 6B (138 isolates; R^2 ^= 0.801; β = -0.907; p < 0.001), 14 (203 isolates; R^2 ^= 0.729; β = -0.871; p = 0.001), 23F (93 isolates; R^2 ^= 0.719; β = -0.866; p = 0.001) and 9V (42 isolates; R^2 ^= 0.559; β = -0.779; p = 0.008). Serotypes 18C (31 isolates) and 4 (9 isolates) did not show significant trends (R^2 ^≤ 0.114; β ≤ -0.393; p ≥ 0.180), probably due to the small number of isolates.

Regarding non-susceptibility, only PCV7 serotypes (as a group) and serotypes 19A and 3 had more than 10 isolates per year and could be analysed in separate. Serotype 3 was fully susceptible to penicillin, amoxicillin and cefotaxime over the study period, with non-susceptibility rates to erythromycin < 10% from 2005 on.

Table 2 shows annual non-susceptibility rates in all middle ear fluid isolates and in the subgroups of 19A and PCV7 isolates. Considering all middle ear fluid isolates, no significant trends were found for non-susceptibility to penicillin (R^2 ^= 0.219; β = -0.468; p = 0.172) or erythromycin (R^2 ^= 0.145; β = -0.381; p = 0.277), with non-susceptibility rates in the range of 36.7% to 58.5% for both antibiotics in the period 2001-2009. Moreover, non-susceptibility rates to cefotaxime were ≤ 8.5% along the study period, showing no trends (R^2 ^= 0.142; β = 0.377; p = 0.238). However, non-susceptibility to amoxicillin showed a significant increasing trend (R^2 ^= 0.528; β = 0.727; p = 0.017), with non-susceptibility rates within the range 6.0% to 8.8% in 2001-5, 10.8% to 19.0% in 2006-7, and 24.1% to 25.0% in 2008-9.

**Table 2:** Number of paediatric middle ear fluid isolates (MEF), and among them, number of PCV-7 and 19A isolates, with per-group percentages of non-susceptibility to penicillin (PEN), amoxicillin (AMX), cefotaxime (CTX), and erythromycin (ERY)

	97-00	2001	2002	2003	2004	2005	2006	2007	2008	2009
**MEF (n)**	481	210	171	159	180	166	176	173	220	141
PEN	69.0	58.1	58.5	42.8	36.7	45.8	41.5	47.4	53.2	51.1
AMX	-	8.1	8.8	8.2	6.7	6.0	10.8	19.0	25.0	24.1
CTX	5.2	4.8	4.1	1.9	6.1	1.8	1.1	6.9	6.8	8.5
ERY	55.3	51.0	51.5	45.3	42.8	48.8	49.4	48.6	52.7	44.7
**PCV-7 (n)**	340	132	109	77	64	40	33	23	22	15
PEN	88.5	82.6	83.5	67.5	68.7	77.5	69.7	73.9	81.8	86.7
AMX	-	12.1	13.8	13.0	17.2	15.0	21.2	26.1	50.0	53.3
CTX	6.5	7.6	6.4	3.9	12.5	2.5	3.0	8.7	9.1	6.7
ERY	65.0	57.6	63.3	58.4	64.1	67.5	63.6	43.5	36.4	53.2
**19A (n)**	32	20	21	27	38	53	55	67	103	50
PEN	50.0	40.0	23.8	44.4	47.4	62.3	67.3	70.1	80.6	84.0
AMX	-	0.0	0.0	7.4	2.6	5.7	21.8	38.8	37.9	38.0
CTX	3.1	0.0	0.0	0.0	7.9	3.8	1.8	14.9	11.7	20.0
ERY	50.0	65.0	33.3	59.3	68.4	79.2	78.2	82.1	85.4	76.0

Non-susceptibility: PEN ≥ 0.12 μg/ml; AMX ≥ 4 μg/ml; CTX ≥ 2 μg/ml; ERY ≥ 0.5 μg/ml

For serotype 19A (for which the prevalence increased over the study period), increasing trends were found for non-susceptibility to penicillin (R^2 ^= 0.726; β = 0.870; p = 0.001), amoxicillin (R^2 ^= 0.804; β = 0.909; p < 0.001), cefotaxime (R^2 ^= 0.546; β = 0.772; p = 0.005) and erythromycin (R^2 ^= 0.546; β = 0.772; p = 0.009). Non-susceptibility rates to penicillin and erythromycin were around 80% in 2008-2009. Non-susceptibility to amoxicillin was first detected in 2003 and increased up to 38.0% in 2009. Non-susceptibility to cefotaxime was found in a single strain in 1999 whereas in the period 2001-2009 was firstly detected in 2004 and increased up to 20.0% in 2009.

When analyzing non-susceptibility rates in PCV7 serotypes (for which the prevalence decreased over the study period), no trends were found for penicillin (R^2 ^= 0.106; β = -0.130; p = 0.721) with non-susceptibility rates of ≥ 67.5%, neither for erythromycin (R^2 ^= 0.283; β = -0.602; p = 0.065) with non-susceptibility rates of ≥ 36.4%, nor for cefotaxime (R^2 ^= 0.120; β = 0.067; p = 0.854) with non-susceptibility rates of ≤ 12.5% over the studied period. In contrast, there was a significant increasing trend for non-susceptibility to amoxicillin (R^2 ^= 0.702; β = 0.858; p = 0.002), with rates around 50% in 2008-9. The individual analysis of PCV7 serotypes to determine serotype(s) responsible(s) for this increasing trend, showed that there was a non-susceptibility increasing trend for serotypes 19F (332 isolates; R^2 ^= 0.517; β = 0.755; p = 0.012), 6B (138 isolates; R^2 ^= 0.690; β = 0.851; p = 0.002), and 9V (49 isolates; R^2 ^= 0.379; β = 0.669; p = 0.034), but not for serotypes 14 (203 isolates), 23F (93 isolates) and 4 (9 isolates). Isolates belonging to serotype 18C (31 isolates) were fully susceptible to amoxicillin.

Considering multiple resistance as a concomitant non-susceptibility event to β-lactams (using cefotaxime as marker) and erythromycin in a single isolate, the percentage of isolates exhibiting multiple resistance was > 10% in serotype 19A, with rates ranging from 11.7% to 20.0% in the 2007-2009 period.

## Discussion

Data from the United States at the time of PCV7 licensure showed that the vaccine (including the most common invasive and often drug-resistant serotypes causing disease in children) provided 89.1% protective efficacy against invasive disease caused by all serotypes [21]. In a post-introduction surveillance study the percentage of reduction in invasive disease caused by PCV7 serotypes was 78%, with 50% reduction in invasive disease caused by vaccine-related serotypes [22]. However, serotype replacement has been reported in surveillances (laboratory-based surveillance system) analyzing invasive isolates along the current decade in Spain [11-13], USA [23] and several European countries [24]. In addition, several studies have shown that PCV7 vaccination also reduces the nasopharyngeal carriage of vaccine serotypes and increases carriage of non-vaccine types, with an overall rate of pneumococcal colonization unchanged in most cases [25-28]. AOM is the most common infection following pneumococcal colonization. Since PCV7 introduction, 57%-66.7% reduction in otitis media episodes caused by PCV7 serotypes has been reported with an increase in cases caused by non-vaccine serotypes, both in clinical trials [21,29] and post-introduction surveillances [30,31]. However the possible decrease in PCV7 serotypes among middle ear fluid isolates after the introduction of the PCV7 in Spain, remained to be explored.

The present study showed that serotype replacement has occurred among middle ear isolates from children in Spain as demonstrated by the decrease in PCV7 serotypes (from 62.9% in 2001 to 10.6% in 2009) and the increase in the prevalence of serotype 19A (from 6.7% in 1997-00 to > 35% from 2007 on) and to a lesser extent of serotype 3 (already described as an emergent serotype due to replacement [32]).

In a previous study analysing invasive isolates from children, one important fact of the significant decrease in the prevalence of PCV7 serotypes (from 62.4% in 2000 to 14.6% in 2007) was that the reduction was followed by a significant decrease in non-susceptibility to penicillin and erythromycin (from 51.4% and 39.5%, respectively, to around 20% for both antibiotics in 2007) [12]. In contrast to this, the main finding of the present study is that in middle ear fluid isolates from children the significant decrease in PCV7 serotypes after PCV7 introduction was not associated with a decrease in non-susceptibility to penicillin or erythromycin among middle ear fluid isolates. High non-susceptibility rates (≈40-50%) to these two antibiotic markers were found all over the study period among all middle ear fluid isolates. Even more, a significant increase in amoxicillin non-susceptibility (from 8.1% in 2001 to ≈25% in 2008 and 2009) was found. By analysing the two major subgroups (serotype 19A and non-PCV7 serotypes), it can be deduced that this increase was mainly attributed to the significant increase in the prevalence of serotype 19A. In this serotype, it is important to remark that the first middle ear isolates non-susceptible to amoxicillin were detected in 2003, and proportions increased up to ≈38% in the period 2007-9, as occurred with non-susceptibility to cefotaxime that in the present decade was first detected in 2004 and reached 20% in 2009. Non-susceptibility to these two β-lactams and to erythromycin (multiple resistance) was found in > 10% of 19A isolates in the last years of the decade. This multiresistance pattern, challenging common therapeutic options, has also been described after vaccine introduction in USA in serotype 19A middle ear fluid isolates from children [33].

However, in our study, amoxicillin non-susceptibility cannot be entirely attributed to serotype 19A or other non-PCV7 serotypes since the significant decrease in PCV7 serotypes was associated with a significant increase in amoxicillin non-susceptibility among them, clustered in PCV7 serotypes 19F, 6B and 9V. The fact that these serotypes exhibited a significant decreasing trend over the study period suggests that these serotypes (and thus non-susceptibility to amoxicillin) will further decrease as vaccination rates increase. Nevertheless, the contribution of PCV7 serotypes to the prevalence of amoxicillin non-susceptibility among all middle ear isolates is relatively low since, although amoxicillin non-susceptibility in PCV7 isolates was ≈50% in 2008-9, all PCV7 isolates represented only ≈10% of all middle ear isolates received in the SRLP. The high antibiotic consumption for the treatment of AOM (the most common infection for which antibiotics are prescribed in children in the community [2]) represents the major pressure for resistance selection [11]. This antibiotic pressure over the selectable residual occurrence of PCV7 serotypes (probably due to low vaccine coverage) may be a reason for the lack of reduction in resistance in parallel to the reduction in the prevalence of PCV7 serotypes (in contrast to invasive isolates). In this sense a previous study showed that *S. pneumoniae *is more often resistant to antibiotics in otitis prone children [31], and consumption of aminopenicillins is by far greater than consumption of macrolides in Spain [11].

As stated, data of this study came from a passive laboratory-based surveillance system with strains sent on a voluntary basis from hospitals all over the Spanish geography. Although the high number of isolates analysed over the large study period and its geographic diversity strengthen conclusions about serotypes involved in otitis in Spain, three main limitations of the study may be identified: 1) isolates were sent by microbiology departments in hospitals, and the SRLP does not have clinical information on the type of otitis or on how samples were collected, 2) isolates came from hospitals, thus they are not necessarily representative of pneumococci causing otitis in children attending primary care centres and 3) multilocus sequence typing was not performed, thus specific sequence types responsible for the increase in 19A cannot be identified, as was done for paediatric invasive isolates in a previous study highlighting the outstanding role of sequence types 320 and 878 (first detected in 2003 and 2007, respectively) [34].

## Conclusions

The high prevalence of serotype 19A among paediatric middle ear isolates and the high rates of amoxicillin non-susceptibility found in this serotype are of great concern since amoxicillin is the most common antibiotic used in the treatment of AOM. These data suggest that non-PCV7 serotypes (mainly serotype 19A followed by serotypes 3 and 6A) are important etiological agents of AOM and support the added value of the broader coverage of the new 13-valent conjugate vaccine. Reducing nasopharyngeal carriage (and subsequently preventing AOM) of vaccine serotypes may be an added value in a context where it has been postulated that the reduction in drug-resistant *S. pneumoniae *will require a combination of conjugate vaccine and reductions in antibiotic use [28].

## Abbreviations

AOM: Acute otitis media; MEF: Middle ear fluid; PCV7: 7-valent pneumococcal conjugate vaccine; SRLP: Spanish reference laboratory for pneumococci.

## Competing interests

AF has received travel grants from Pfizer S.A. for attending congresses to present studies supported in part by unrestricted grants from Pfizer S.A. Other authors declare that they have no competing interests.

## Authors' contributions

Conceived and designed the experiments: AF, LA, M-JG. Performed the experiments: M-DV, OR. Analyzed the data: LA, M-JG, J-JG. Wrote the paper: LA, M-JG. Reviewed and approved the manuscript: AF, M-DV, OR, J-JG. All authors read and approved the final manuscript.

## Pre-publication history

The pre-publication history for this paper can be accessed here:

http://www.biomedcentral.com/1471-2334/11/239/prepub
